# Dataset of the immersion enthalpy of activated carbons chemically modified in methylparaben aqueous solution: Relation with adsorption

**DOI:** 10.1016/j.dib.2019.104100

**Published:** 2019-06-08

**Authors:** Astrid R. Moreno-Marenco, Liliana Giraldo, Juan Carlos Moreno-Piraján

**Affiliations:** a Departamento de Química, Facultad de Ciencias, Universidad Nacional de Colombia, Sede Bogotá, Carrera 30 No. 45-03 Bogotá, Colombia; b Departamento de Química, Facultad de Ciencias, Universidad de los Andes, Carrera, 1 No. 18 A-12 Bogotá, Colombia

**Keywords:** Immersion calorimetry, Adsorption, Methylparaben

## Abstract

Methylparaben (MePB) is a type of emerging contaminant [1], commonly present in wastewater and surface water, widely used as preservatives in personal care products. The risk associated with the presence of this pollutant in the environment is due to their classification as an endocrine disruptor [2]. Two activated carbons obtained from African palm shell (Elaeis Guineensis) modified chemically by impregnation with CaCl_2_ (GC1) and MgCl_2_ (GM1) at 1% wt/v and carbonized in CO_2_ atmosphere at 1173 K, were prepared. The process of adsorption of methylparaben from aqueous solution to activated carbon is due to the interactions between the adsorbate and the adsorbent, which can be quantified through the determination of immersion enthalpies in aqueous solutions of MePB, finding values of - 22.45 and −45.23 J g^−1^ for the immersion in the solution of 100 mg L^−1^, -3.31 and −44.02 J g^−1^ for the immersion in the solution of 200 mg L^−1^ and -21.31 and −54.51 J g^−1^, showing the highest values carbon GC1. To evaluate the efficiency of MePB adsorption on the obtained solids, Langmuir and Freundlich adsorption models were determined in order to relate the quantities adsorbed with the immersion enthalpies and know the intensity of the energy interactions between the MePB and the activated carbons.

**Table:** Specifications Table

Subject area	*Physical chemistry*
More specific subject area	*Adsorption and Thermodynamic*
Type of data	*Table, figure*
How data was acquired	*Immersion calorimeter (local construction), spectrophotometer UV-Vis (ThermoElectron Genesys 10uv)*
Data format	*Raw and analyzed.*
Experimental factors	*A raw of activated carbons were prepared from African palm shells by chemical activation with CaCl*_*2*_*(GC1) and MgCl*_*2*_*(GM1) at 1% w/v and physical activation with CO*_*2*_*at* 1173 *K.*
Experimental features	*The experiments of methylparaben adsorption were done in batch conditions. To determinate the immersion enthalpy the activated carbons were immersed in water and methylparaben aqueous solutions, respectively. An electric calibration was performed.*
Data source location	*Chemistry Department, Faculty of Sciences. Universidad Nacional de Colombia. Bogotá, Colombia.*
Data accessibility	*Data are provided in this article*
Related research article	Astrid R. Moreno-Marenco, Liliana Giraldo, Juan Carlos Moreno-Piraján. *Dataset of the immersion enthalpy of activated carbons chemically modified in methylparaben aqueous solution: Relation with adsorption.*

**Table:** 

**Value of the data** • Data obtained by immersion calorimetry provides an insight to adsorbent-adsorbate interactions stablished between activated carbons chemically modified with metallic salts for methylparaben adsorption. This data is related to the adsorption capacity showed that the modification of the activated carbons affects their ability to adsorption. • The intensity of the interactions can be evaluated and compared by testing another type of solids and immersion liquids. • The information obtained can be used to complete the data on the emerging contaminants adsorption from aqueous solutions

## 1 Data

**Fig. 1:**
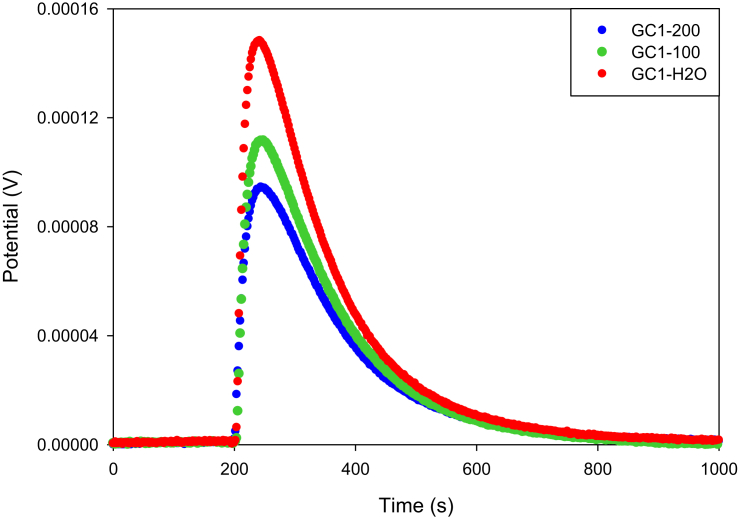
Calorimetric curves of the immersion of GC1 into water (GC1-H_2_O) and MePB solutions at 100 mg L^−1^ (GC1-100) and 200 mg L^−1^ (GC1-200) at 18 °C.

**Fig. 2:**
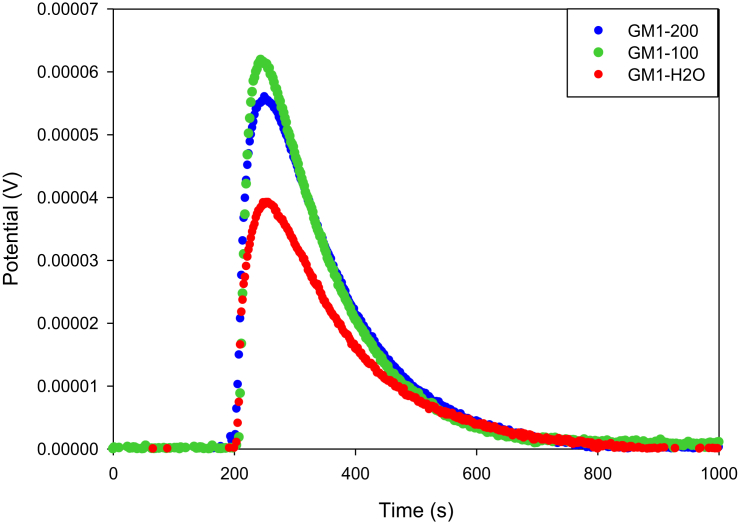
Calorimetric curves of the immersion of GM1 into water (GM1-H_2_O) and MePB solutions at 100 mg L^−1^ (GM1-100) and 200 mg L^−1^ (GM1-200) at 18 °C.

**Fig. 3:**
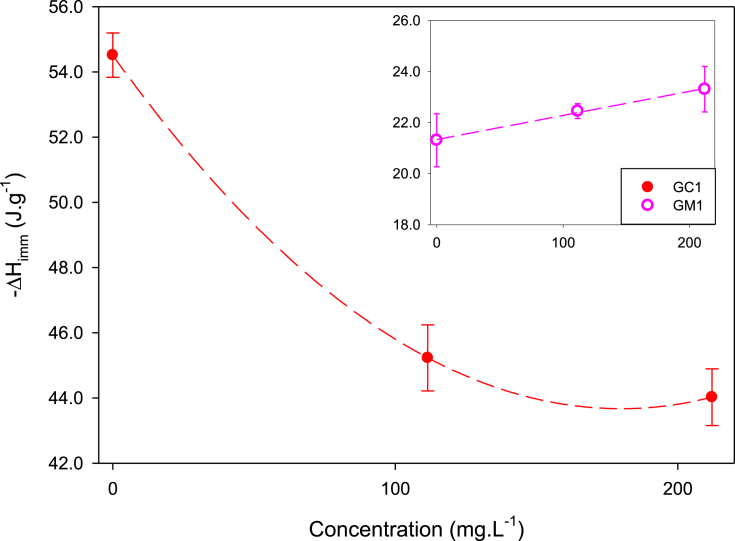
Relation between the concentration of MePB with immersion enthalpy of the activated carbons.

**Fig. 4:**
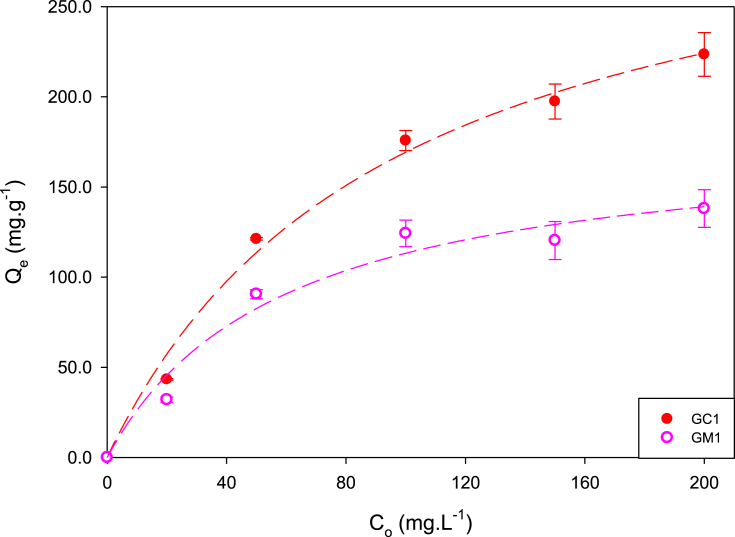
Methylparaben adsorption isotherms at 18 °C.

**Fig. 5:**
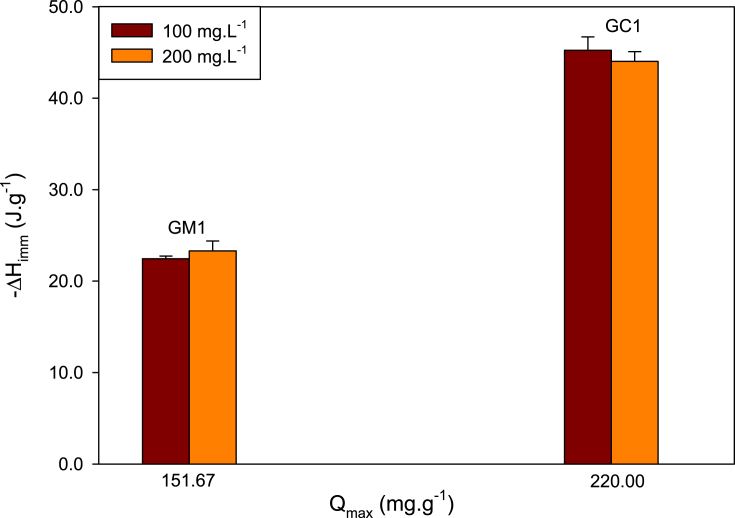
Relation between adsorption capacity with immersion enthalpy of activated carbons.

These data correspond to calorimetric curves obtained by immersion calorimetry, for two activated carbons chemically modified with CaCl_2_ (GC1) in Fig. 1 and MgCl_2_ (GM1) in Fig. 2 at 1% w/v, respectively and carbonized in CO_2_ flow at 1173 into water and methylparaben aqueous solutions (MePB) of 100 and 200 mg L^−1^ and their corresponding immersion values at 18 °C are presented in Table 1. Fig. 3 shows the relation between the concentration of MePB with immersion enthalpy of the activated carbons. Fig. 4 presents the data of adsorption study carried out using activated carbons prepared previously with MePB solutions at 18 °C. Table 2 presents the corresponding data obtained in MePB adsorption. Fig. 5 shows the relation between adsorption capacity with immersion enthalpy of activated carbons.

**Table 1:** Values of immersion enthalpy of activated carbons into water and methylparaben solutions at 100 mg L^−1^ and 200 mg L^−1^ at 18 °C.

GAC	$-\Delta {\text{H}}_{{\text{imm}\;\text{H}}_{2}\text{O}}$(J.g^−1^)	$-\Delta {\text{H}}_{\text{imm}\;\text{MePB}100}$(J.g^−1^)	$-\Delta {\text{H}}_{\text{imm}\;\text{MePB}200}$ (J.g^−1^)
GC1	54.51 ± 0.68	45.23 ± 1.47	44.02 ± 1.07
GM1	21.31 ± 1.14	22.45 ± 0.29	23.31 ± 1.09

**Table 2:** Constants of isotherm models for adsorption of MePB onto activated carbons dosage of 5 mg L^−1^ at 18 °C.

GAC	Langmuir $q_{e}=\frac{Q_{max}K_{L}C_{e}}{1+K_{L}C_{e}}$			Freundlich $q_{e}=K_{F}C_{e}^{1/nF}$		
	Q_max_ (mg.g^−1^)	K_L_ (L.mg^−1^)	r^2^	K_F_ (mg.g^−1^) (L.mg^−1^)^n−1^	n_F_	r^2^
GC1	220.00 ± 3.37	0.16	0.947	58.31 ± 0.62	3.48	0.937
GM1	151.67 ± 3.54	0.056	0.948	29.32 ± 0.086	5.31	0.901

## 2 Experimental design, materials and methods

### 2.1 Preparation of activated carbons

Activated carbons were prepared from African palm shell sieved to particle size of 4 mm and impregnant with solutions of CaCl_2_ (GC1) and MgCl_2_ (GM1) at 1% w/v, respectively in a thermostat bath at 358 K for 48 h. After that the samples were carbonized in CO_2_ flow at 1173 K for 6h. Finally, it was washed with HCl 5% and water to remove remain salts and dried for 24 h at 363 K.

### 2.2 Immersion enthalpy determination

Immersion calorimetry took place in a heat conduction microcalorimeter (local construction) [1], [2], [3] with a cell in stainless steel in which 10 mL of the immersion liquid was placed, using water and MePB aqueous solutions of 100 and 200 mg L^−1^ at 18 °C. The capture of the output electric potential started and the stabilization of the calorimeter until base line was achieved. Around 100 mg of each activated carbon was weighed into a glass ampoule with a fragile peak and the immersion of the sample into the liquid was performed. The resulting thermal changes were registered until baseline is stabilized again and finally, an electric calibration was done [4], [5].

The results of the immersion enthalpy of the activated carbons in water and MePB solutions are shown in Table 1. High values of immersion enthalpies of activated carbons were obtained for sample GC1 exhibited a greater interaction because the thermal effect that developed when this carbon was immersed. Considering the order of magnitude of the enthalpic values of the adsorbent–adsorbate interaction, this may be considered a physisorption [6].

Fig. 1 shows the calorimetric curves of the immersion of GC1 into water and MePB solutions of 100 y 200 mg L^−1^ showing that this carbon has a greater interaction with water and less interaction with the MEPB solutions since the area of the peak under the curve is smaller for the immersion in the solutions, indicating that the process is becoming increasingly endothermic as the concentration of MePB increases [7]. On the other hand, in Fig. 2 there is a lower interaction with water and a greater interaction as the concentration of the solutions increases, indicating that the process becomes more exothermic for the carbon GM1, as shown in Fig. 3.

### 2.3 Methylparaben adsorption

Adsorption studies were carried out using 25 mg of activated carbons prepared previously, with 50 mL of methylparaben solutions (MePB) Alfa Aesar analytic grade in the range from 20 to 200 mg L^−1^ for 3 weeks at 18 °C to ensure the system reached equilibrium. The residual concentration of MePB was measured by UV-Vis Spectroscopy at 254 nm.

Fig. 4 presents the adsorption isotherms of MePB at 18 °C. Experimental data obtained in MePB adsorption were fit to Langmuir and Freundlich models, which are presented in Table 2, showing a better fit with Langmuir model to both carbons. Also evidenced that carbon GC1 presents a higher adsorption of with a maximum adsorption of 220.00 ± 3.37 mg g^−1^, which is in accordance with the greater immersion enthalpy that this carbon presents, as displayed in Fig. 5 that shows the relation between adsorption capacity with immersion enthalpy of activated carbons.
